# Supplementary material for: Pushing the envelope: Micro-transmitter effects on small juvenile Chinook salmon (Oncorhynchus tshawytscha)
Source: PLoS One. 2020 Mar 25;15(3):e0230100. doi: 10.1371/journal.pone.0230100 (PMC7094837; doi:10.1371/journal.pone.0230100)
Supplement: S1 Table — Specifications of acoustic tags (Juvenile Salmonid Acoustic Telemetry System transmitters, or JSAT) and passive integrated transponder tags (SST tag TX-1411SST) used in both study years. (DOCX) [file pone.0230100.s001.docx]

**S1 Table. Tag specifications.** Specifications of acoustic tags (Juvenile Salmonid Acoustic Telemetry System transmitters, or JSAT) and passive integrated transponder tags (SST tag TX‑1411SST) used in both study years.

|  |  | | |
| --- | --- | --- | --- |
|  | Acoustic tags | | Passive integrated transponder tags  2007 and 2008 |
| Mean | 2007 | 2008 |  |
| Length (mm) | 16.10 | 12.00 | 12.48 |
| Height (mm) | 4.10 | 3.50 |  |
| Width (mm) | 5.90 | 5.30 |  |
| Diameter (mm) |  |  | 2.07 |
| Weight in air (g) | 0.60 | 0.42 | 0.10 |
| Mass in water (g) | 0.38 | 0.30 |  |
| Volume (mL) | 0.24 | 0.14 |  |
|  |  |  |  |
|  |  |  |  |
