# Supplementary material for: Pushing the envelope: Micro-transmitter effects on small juvenile Chinook salmon (Oncorhynchus tshawytscha)
Source: PLoS One. 2020 Mar 25;15(3):e0230100. doi: 10.1371/journal.pone.0230100 (PMC7094837; doi:10.1371/journal.pone.0230100)
Supplement: S1 Appendix — (DOCX) [file pone.0230100.s002.docx]

**S1 Appendix: Downstream survival**

**Table A in S1 Appendix. Estimated survival to Snake River dams for yearlings, 2007**. Mean probability of survival to each downstream dam on the Snake River for tag treatment groups of yearling Chinook released at Lower Granite Dam. Geometric mean survival ratios (AT/PIT) from each downstream detection location were compared using *t*‑tests.

|  |  |  | |  |  |
| --- | --- | --- | --- | --- | --- |
| Release date | **Mean estimated survival to Snake River dams for yearling Chinook salmon, 2007 (SE)** | | | *t* | *P* |
|  | Acoustic transmitter  (AT) | | Passive integrated transponder (PIT) |  |  |
|  |  | |  |  |  |
|  | Little Goose Dam (60 km) | | | | |
| 25 Apr | 0.87 (0.05) | | 0.96 (0.03) |  |  |
| 26 Apr | 0.88 (0.06) | | 0.96 (0.03) |  |  |
| 28 Apr | 0.95 (0.06) | | 0.92 (0.03) |  |  |
| 1 May | 0.90 (0.06) | | 0.91 (0.03) |  |  |
| 3 May | 0.95 (0.09) | | 0.95 (0.03) |  |  |
| 5 May | 1.02 (0.09) | | 0.85 (0.03) |  |  |
| 9 May | 0.92 (0.04) | | 0.91 (0.02) |  |  |
| 10 May | 0.93 (0.04) | | 0.90 (0.02) |  |  |
| 12 May | 0.91 (0.05) | | 0.95 (0.02) |  |  |
| 15 May | 0.93 (0.05) | | 0.99 (0.03) |  |  |
| Total | 0.93 (0.01) | | 0.93 (0.01) | 0.14 | 0.893 |
|  |  | |  |  |  |
|  | Lower Monumental Dam (106 km) | | | | |
| 25 Apr | 0.88 (0.05) | | 0.85 (0.02) |  |  |
| 26 Apr | 0.87 (0.06) | | 0.82 (0.02) |  |  |
| 28 Apr | 0.97 (0.08) | | 0.88 (0.03) |  |  |
| 1 May | 0.84 (0.07) | | 0.84 (0.03) |  |  |
| 3 May | 1.15 (0.22) | | 0.94 (0.05) |  |  |
| 5 May | 0.90 (0.05) | | 0.88 (0.03) |  |  |
| 9 May | 0.87 (0.04) | | 0.84 (0.02) |  |  |
| 10 May | 0.92 (0.05) | | 0.91 (0.02) |  |  |
| 12 May | 1.01 (0.13) | | 0.93 (0.04) |  |  |
| 15 May | 0.82 (0.05) | | 0.89 (0.03) |  |  |
| Total | 0.92 (0.03) | | 0.88 (0.01) | 1.98 | 0.08 |
|  |  | |  |  |  |
|  | Ice Harbor Dam (157 km) | | | | |
| 25 Apr | 0.73 (0.02) | | 0.81 (0.03) |  |  |
| 26 Apr | 0.77 (0.07) | | 0.82 (0.04) |  |  |
| 28 Apr | 0.80 (0.07) | | 0.90 (0.06) |  |  |
| 1 May | 0.68 (0.06) | | 0.85 (0.06) |  |  |
| 3 May | 0.76 (0.10) | | 0.91 (0.05) |  |  |
| 5 May | 0.87 (0.06) | | 0.83 (0.03) |  |  |
| 9 May | 0.88 (0.10) | | 0.81 (0.06) |  |  |
| 10 May | 0.96 (0.14) | | 0.89 (0.06) |  |  |
| 12 May | 0.81 (0.12) | | 0.81 (0.04) |  |  |
| 15 May | 0.87 (0.15) | | 0.80 (0.07) |  |  |
| Total | 0.81 (0.03) | | 0.84 (0.01) | 1.14 | 0.285 |
|  |  | |  |  |  |

**Table B in S1 Appendix. Estimated survival to Columbia River Dams for yearlings, 2007**. Mean probability of survival to downstream dams on the Columbia River for tag treatment groups of yearling Chinook released at Lower Granite Dam. Geometric mean survival ratios (AT/PIT) from each downstream detection location were compared using *t*‑tests.

|  |  |  | |  |  |
| --- | --- | --- | --- | --- | --- |
| Release date | **Mean estimated survival to Columbia River dams for yearling Chinook salmon, 2007 (SE)** | | |  | |
|  | Acoustic transmitter  (AT) | | Passive integrated transponder (PIT) | *t* | *P* |
|  |  | |  |  |  |
|  | McNary Dam (225 km) | | | | |
| 25 Apr | 0.76 (0.03) | | 0.81 (0.02) |  |  |
| 26 Apr | 0.70 (0.03) | | 0.81 (0.02) |  |  |
| 28 Apr | 0.76 (0.03) | | 0.76 (0.02) |  |  |
| 1 May | 0.65 (0.03) | | 0.82 (0.02) |  |  |
| 3 May | 0.64 (0.03) | | 0.83 (0.02) |  |  |
| 5 May | 0.77 (0.03) | | 0.79 (0.02) |  |  |
| 9 May | 0.75 (0.03) | | 0.75 (0.03) |  |  |
| 10 May | 0.78 (0.03) | | 0.74 (0.02) |  |  |
| 12 May | 0.69 (0.03) | | 0.73 (0.02) |  |  |
| 15 May | 0.74 (0.03) | | 0.72 (0.02) |  |  |
| Total | 0.72 (0.02) | | 0.78 (0.01) | 0.14 | 0.893 |
|  |  | |  |  |  |
|  | John Day Dam (348 km) | | | | |
| 25 Apr | 0.68 (0.04) | | 0.70 (0.03) |  |  |
| 26 Apr | 0.62 (0.03) | | 0.74 (0.04) |  |  |
| 28 Apr | 0.63 (0.04) | | 0.71 (0.04) |  |  |
| 1 May | 0.51 (0.04) | | 0.81 (0.05) |  |  |
| 3 May | 0.54 (0.04) | | 0.78 (0.03) |  |  |
| 5 May | 0.68 (0.04) | | 0.80 (0.04) |  |  |
| 9 May | 0.60 (0.03) | | 0.72 (0.05) |  |  |
| 10 May | 0.67 (0.05) | | 0.64 (0.03) |  |  |
| 12 May | 0.59 (0.06) | | 0.76 (0.05) |  |  |
| 15 May | 0.62 (0.05) | | 0.58 (0.04) |  |  |
| Total | 0.62 (0.02) | | 0.72 (0.02) | 3.25 | 0.01 |
|  |  | |  |  |  |
|  | Bonneville Dam (460 km) | | | | |
| 25 Apr | 0.56 (0.03) | | 0.79 (0.20) |  |  |
| 26 Apr | 0.52 (0.04) | | 0.76 (0.24) |  |  |
| 28 Apr | 0.52 (0.04) | | 0.55 (0.15) |  |  |
| 1 May | 0.47 (0.07) | | 0.58 (0.14) |  |  |
| 3 May | 0.48 (0.04) | | 0.63 (0.10) |  |  |
| 5 May | 0.53 (0.03) | | 0.63 (0.12) |  |  |
| 9 May | 0.50 (0.03) | | 0.71 (0.21) |  |  |
| 10 May | 0.52 (0.05) | | 0.64 (0.15) |  |  |
| 12 May | 0.43 (0.03) | | 0.61 (0.17) |  |  |
| 15 May | 0.45 (0.06) | | 0.39 (0.09) |  |  |
| Total | 0.50 (0.01) | | 0.63 (0.04) | 1.14 | 0.285 |
|  |  | |  |  |  |

**Table C in S1 Appendix. Estimated survival to Snake River dams for yearlings, 2008**. Mean probability of survival and relative survival from release to downstream dams on the Snake River for tag treatment groups of yearling Chinook released at Lower Granite Dam. Geometric mean survival ratios (AT/PIT) from each downstream detection location were compared using *t*‑tests.

|  |  | | | |  |
| --- | --- | --- | --- | --- | --- |
| Release  date | **Mean estimated survival to Snake River dams for yearling Chinook salmon, 2008 (SE)** | | | | Relative  survival  (AT/PIT) |
|  | Acoustic transmitter  (AT) | Passive integrated transponder (PIT) | *t* | *P* |  |
|  |  |  |  |  |  |
|  | Little Goose Dam (60 km) | | | | |
| 24 Apr | 0.85 (0.04) | 0.97 (0.04) |  |  | 0.88 (0.05) |
| 29 Apr | 0.96 (0.05) | 0.97 (0.03) |  |  | 1.00 (0.06) |
| 1 May | 0.92 (0.04) | 0.95 (0.02) |  |  | 0.97 (0.04) |
| 3 May | 0.94 (0.04) | 0.96 (0.02) |  |  | 0.98 (0.04) |
| 6 May | 0.90 (0.04) | 0.97 (0.02) |  |  | 0.93 (0.04) |
| 8 May | 0.96 (0.04) | 0.94 (0.01) |  |  | 1.03 (0.04) |
| 10 May | 0.89 (0.03) | 0.94 (0.02) |  |  | 0.94 (0.04) |
| 13 May | 0.88 (0.03) | 0.91 (0.02) |  |  | 0.98 (0.03) |
| 15 May | 0.94 (0.03) | 0.95 (0.02) |  |  | 1.00 (0.04) |
| 17 May | 1.00 (0.03) | 0.96 (0.03) |  |  | 1.05 (0.04) |
| Total | 0.92 (0.01) | 0.95 (0.01) | 1.79 | 0.107 | 0.97 (0.02) |
|  |  |  |  |  |  |
|  | Lower Monumental Dam (106 km) | | | | |
| 24 Apr | 0.93 (0.07) | 0.84 (0.05) |  |  | 1.12 (0.11) |
| 29 Apr | 0.86 (0.04) | 0.98 (0.04) |  |  | 0.87 (0.05) |
| 1 May | 0.90 (0.05) | 0.91 (0.03) |  |  | 1.00 (0.06) |
| 3 May | 0.83 (0.05) | 0.9 (0.02) |  |  | 0.91 (0.06) |
| 6 May | 0.81 (0.05) | 0.97 (0.03) |  |  | 0.83 (0.06) |
| 8 May | 0.92 (0.09) | 0.98 (0.04) |  |  | 0.94 (0.10) |
| 10 May | 0.81 (0.06) | 0.94 (0.05) |  |  | 0.86 (0.08) |
| 13 May | 0.89 (0.04) | 0.89 (0.04) |  |  | 1.00 (0.06) |
| 15 May | 0.89 (0.03) | 0.89 (0.03) |  |  | 1.01 (0.05) |
| 17 May | 0.97 (0.03) | 0.98 (0.04) |  |  | 0.99 (0.05) |
| Total | 0.88 (0.02) | 0.93 (0.02) | 1.86 | 0.096 | 0.95 (0.03) |
|  |  |  |  |  |  |
|  | Ice Harbor Dam (157 km) | | | | |
| 24 Apr | 0.75 (0.06) | 0.68 (0.04) |  |  | 1.10 (0.11) |
| 29 Apr | 0.76 (0.04) | 0.85 (0.04) |  |  | 0.89 (0.06) |
| 1 May | 0.83 (0.05) | 0.81 (0.03) |  |  | 1.02 (0.08) |
| 3 May | 0.74 (0.05) | 0.86 (0.03) |  |  | 0.87 (0.06) |
| 6 May | 0.70 (0.06) | 0.86 (0.04) |  |  | 0.82 (0.08) |
| 8 May | 0.72 (0.03) | 0.90 (0.06) |  |  | 0.80 (0.06) |
| 10 May | 0.88 (0.10) | 0.86 (0.06) |  |  | 1.01 (0.13) |
| 13 May | 0.93 (0.06) | 0.82 (0.06) |  |  | 1.14 (0.12) |
| 15 May | 0.84 (0.05) | 0.78 (0.05) |  |  | 1.08 (0.10) |
| 17 May | 0.83 (0.03) | 0.86 (0.06) |  |  | 0.95 (0.08) |
| Total | 0.80 (0.02) | 0.83 (0.02) | 1.02 | 0.336 | 0.96 (0.04) |
|  |  |  |  |  |  |

**Table D in S1 Appendix. Estimated survival to Columbia River dams for yearlings, 2008**. Mean survival probability and relative survival from release to downstream dams on the Columbia River for tag treatment groups of yearling Chinook salmon released to the tailrace of Lower Granite Dam, 2008. Geometric mean survival ratios (AT/PIT) from each downstream detection location were compared using *t*‑tests.

| Release Date | |  | | | | | Relative survival  (AT/PIT) |
| --- | --- | --- | --- | --- | --- | --- | --- |
|  |  | **Mean estimated survival to Columbia River dams for yearling Chinook salmon, 2008 (SE)** | | | | |  |
|  |  | Acoustic transmitter  (AT) | | Passive integrated transponder (PIT) | *t* | *P* |  |
|  | McNary Dam (225 km) | | | | | | |
| 24 Apr | 0.62 (0.03) | | 0.73 (0.06) | |  |  | 0.85 (0.08) |
| 29 Apr | 0.7 (0.03) | | 0.75 (0.03) | |  |  | 0.93 (0.06) |
| 1 May | 0.71 (0.03) | | 0.79 (0.03) | |  |  | 0.89 (0.05) |
| 3 May | 0.62 (0.03) | | 0.84 (0.03) | |  |  | 0.74 (0.04) |
| 6 May | 0.59 (0.03) | | 0.77 (0.03) | |  |  | 0.77 (0.05) |
| 8 May | 0.63 (0.03) | | 0.78 (0.04) | |  |  | 0.81 (0.05) |
| 10 May | 0.61 (0.04) | | 0.67 (0.05) | |  |  | 0.91 (0.09) |
| 13 May | 0.71 (0.02) | | 0.57 (0.05) | |  |  | 1.25 (0.12) |
| 15 May | 0.76 (0.02) | | 0.78 (0.08) | |  |  | 0.98 (0.11) |
| 17 May | 0.87 (0.06) | | 0.78 (0.08) | |  |  | 1.12 (0.14) |
| Total | 0.68 (0.03) | | 0.75 (0.02) | | 1.87 | 0.095 | 0.91 (0.05) |
|  |  | |  | |  |  |  |
|  | John Day Dam (348 km) | | | | | | |
| 24 Apr | 0.51 (0.05) | | 0.89 (0.21) | |  |  | 0.58 (0.02) |
| 29 Apr | 0.63 (0.05) | | 0.65 (0.07) | |  |  | 0.98 (0.13) |
| 1 May | 0.56 (0.03) | | 0.82 (0.08) | |  |  | 0.69 (0.08) |
| 3 May | 0.47 (0.03) | | 0.9 (0.09) | |  |  | 0.52 (0.06) |
| 6 May | 0.47 (0.03) | | 0.91 (0.11) | |  |  | 0.52 (0.07) |
| 8 May | 0.54 (0.04) | | 0.95 (0.15) | |  |  | 0.57 (0.10) |
| 10 May | 0.59 (0.05) | | 0.69 (0.1) | |  |  | 0.85 (0.14) |
| 13 May | 0.69 (0.05) | | 0.87 (0.13) | |  |  | 0.80 (0.14) |
| 15 May | 0.75 (0.06) | | 0.69 (0.09) | |  |  | 1.09 (0.17) |
| 17 May | 0.79 (0.06) | | 0.91 (0.15) | |  |  | 0.87 (0.16) |
| Total | 0.60 (0.04) | | 0.83 (0.03) | | 4.53 | 0.001 | 0.72 (0.06) |
|  |  | |  | |  |  |  |
|  | Bonneville Dam (460 km) | | | | | | |
| 24 Apr | 0.39 (0.03) | | 0.62 (0.41) | |  |  | 0.63 (0.41) |
| 29 Apr | 0.67 (0.11) | | 0.61 (0.16) | |  |  | 1.09 (0.33) |
| 1 May | 0.49 (0.02) | | 0.42 (0.07) | |  |  | 1.18 (0.21) |
| 3 May | 0.43 (0.04) | | 0.42 (0.07) | |  |  | 1.04 (0.20) |
| 6 May | 0.42 (0.02) | | 0.59 (0.23) | |  |  | 0.71 (0.27) |
| 8 May | 0.52 (0.06) | | 0.44 (0.24) | |  |  | 1.19 (0.67) |
| 10 May | 0.46 (0.03) | | 0.86 (0.59) | |  |  | 0.53 (0.37) |
| 13 May | 0.57 (0.02) | | 0.84 (0.57) | |  |  | 0.69 (0.47) |
| 15 May | 0.65 (0.04) | | 1.25 (1.22) | |  |  | 0.52 (0.51) |
| 17 May | 0.62 (0.04) | | 1.43 (1.4) | |  |  | 0.43 (0.42) |
| Total | 0.52 (0.03) | | 0.75 (0.11) | | 2.79 | 0.021 | 0.75 (0.09) |
|  |  | | |  |  |  |  |
