# Supplementary material for: Pushing the envelope: Micro-transmitter effects on small juvenile Chinook salmon (Oncorhynchus tshawytscha)
Source: PLoS One. 2020 Mar 25;15(3):e0230100. doi: 10.1371/journal.pone.0230100 (PMC7094837; doi:10.1371/journal.pone.0230100)
Supplement: S2 Appendix — (DOCX) [file pone.0230100.s003.docx]

**S2 Appendix: Travel time**

**Fig A in S2 Appendix. Downstream travel time of yearlings, 2007.** Average median travel time to downstream dams on the Snake and Columbia River for acoustic transmitter (AT) and passive integrated transponder (PIT) tag groups of yearling Chinook from release at Lower Granite Dam to detection at dams 60‑460 km downstream. The difference between travel times for treatment groups to John Day was significant. Whisker bars denote standard errors.

**Fig B in S2 Appendix. Downstream travel time of yearlings, 2008.** Average median travel time to downstream dams on the Snake and Columbia River for acoustic transmitter (AT) and passive integrated transponder (PIT) tag groups of yearling Chinook from release at Lower Granite Dam to detection at dams 60‑460 km downstream. The difference between travel times for treatment groups to John Day was significant. (α = 0.05). Whisker bars denote standard errors.

b.)

a.)

Release week

5 June

12 June

19 June

26 June

3 July

12 July

Travel time (days)

0

5

10

15

20

25

30

AT

PIT

= 39.8

Release week

5 June

12 June

19 June

26 June

3 July

12 July

Travel time (days)

0

10

20

30

40

50

AT

PIT

*

*

c.)

d.)

Release week

5 June

12 June

19 June

26 June

3 July

12 July

Travel time (days)

0

5

10

15

20

25

30

35

40

50

AT

PIT

*

Release week

5 June

12 June

19 June

26 June

3 July

12 July

0

10

20

30

40

50

AT

PIT

*

*

Travel time (days)

**Fig C in S2 Appendix. Downstream travel time of subyearlings, 2007.** Median travel time for acoustic transmitter (AT) and passive integrated transponder (PIT) tag treatment replicate groups of subyearlings by week of release. Travel time was calculated as time between release and detection at a. Little Goose (60 km), b. Lower Monumental (106 km), c. Ice Harbor (157 km), and d. McNary Dam (225 km). Error bars represent 10th and 90th percentiles arriving at each location. Asterisks denote release groups where detections were too low to calculate an estimate.
