# Supplementary material for: Pushing the envelope: Micro-transmitter effects on small juvenile Chinook salmon (Oncorhynchus tshawytscha)
Source: PLoS One. 2020 Mar 25;15(3):e0230100. doi: 10.1371/journal.pone.0230100 (PMC7094837; doi:10.1371/journal.pone.0230100)
Supplement: S3 Appendix — (DOCX) [file pone.0230100.s004.docx]

**S3 Appendix: Gross necropsy**

**Table A in S3 Appendix. Scoring criteria for gross necropsy.** Geode index for gross necropsy of Chinook salmon reference and tag treatment groups released and recaptured downstream in 2007 and 2008.

|  |  | | | | | |
| --- | --- | --- | --- | --- | --- | --- |
|  | Score number and definition | | | | | |
|  | 0 | 1 | 2 | 3 | 4 | 5 |
| Smolt Index | fully smolted | partially smolted | weakly smolted | non- smolted | N/A | N/A |
|  |  |  |  |  |  |  |
| Eyes | normal | diminutive | hemorrhagic | exopthalmic | cataract | blind/missing |
|  |  |  |  |  |  |  |
| Fins | normal | opaque | frayed | clubbed/missing | N/A | N/A |
|  |  |  |  |  |  |  |
| Gills | normal | pale | marginate | clubbed | N/A | N/A |
|  |  |  |  |  |  |  |
| Pseudobranchs | normal | swollen | lithic | swollen & lithic | inflamed | N/A |
|  |  |  |  |  |  |  |
| Caecal Fat | none | <50% caeca covered | 50% caeca covered | > 50% caeca covered | entire caeca covered | N/A |
|  |  |  |  |  |  |  |
| Spleen | red | black | enlarged | granular | nodular | black enlarged |
|  |  |  |  |  |  |  |
| Food in Stomach | absent | present | N/A | N/A | N/A | N/A |
|  |  |  |  |  |  |  |
| Hind gut | no inflammation | mild inflammation | severe inflammation | N/A | N/A | N/A |
|  |  |  |  |  |  |  |
| Liver | normal/firm/ red‑brown | slight discoloration | pale | fatty/ pale‑brown  /greasy | nodules in liver | focal discoloration |
| Gall Bladder | yellow/empty/  partially full | yellow/full/  distended | light/  grass green | dark green/  blue‑green |  | N/A |
|  |  |  |  |  |  |  |
| Kidney | normal | pale | swollen | mottled | granular | N/A |
|  |  |  |  |  |  |  |
|  |  |  |  |  |  |  |

**Table B in S3 Appendix. Summary of gross necropsy scores for yearling Chinook**. Score summary for yearling Chinook salmon tagged, released, and recaptured during migration in 2007 and 2008. Numbers represent the proportion of study fish from each reference or tag treatment group that fell into each Geode index category (observations binned into normal vs. non-normal summary scores).

|  |  |  | | | | | |  |  | | | | | |
| --- | --- | --- | --- | --- | --- | --- | --- | --- | --- | --- | --- | --- | --- | --- |
| Metric | Score summary | 2007^*^ | | | | | |  | 2008^*^ | | | | | |
|  |  | Reference | McNary Dam (225 km) | |  | Bonneville Dam (460 km) | |  | Reference | McNary Dam (225 km) | |  | Bonneville Dam (460 km) | |
|  |  |  | AT | PIT |  | AT | PIT |  |  | AT | PIT |  | AT | PIT |
|  |  |  |  |  |  |  |  |  |  |  |  |  |  |  |
| Smolt Index | Fully smolted (0) | 50 | 97 | 98 |  | 58 | 70 |  | 74 | 95 | 98 |  | 99 | 97 |
|  | Partially or non-smolted (1‑3) | 50 | 2 | 2 |  | 43 | 31 |  | 26 | 5 | 2 |  | 1 | 3 |
|  |  |  |  |  |  |  |  |  |  |  |  |  |  |  |
| Eyes | Normal (0) | 100 | 95 | 98 |  | 98 | 100 |  | 99 | 85 | 97 |  | 99 | 99 |
|  | Abnormality or damage (1-5) | 0 | 5 | 2 |  | 2 | 0 |  | 1 | 15 | 3 |  | 1 | 1 |
|  |  |  |  |  |  |  |  |  |  |  |  |  |  |  |
| Fins | Normal (0) | 90 | 68 | 73 |  | 94 | 95 |  | 94 | 10 | 21 |  | 91 | 87 |
|  | Opaque or damaged (1-3) | 10 | 33 | 26 |  | 7 | 5 |  | 6 | 90 | 79 |  | 9 | 13 |
|  |  |  |  |  |  |  |  |  |  |  |  |  |  |  |
| Gills | Normal (0) | 100 | 100 | 98 |  | 100 | 100 |  | 99 | 97 | 97 |  | 98 | 98 |
|  | Pale, marginate or clubbed (1‑3) | 0 | 0 | 2 |  | 0 | 0 |  | 1 | 3 | 3 |  | 2 | 2 |
|  |  |  |  |  |  |  |  |  |  |  |  |  |  |  |
| Pseudobranchs | Normal (0) | 100 | 100 | 100 |  | 100 | 100 |  | 100 | 99 | 100 |  | 100 | 100 |
|  | Swollen, lithic, inflamed (1‑4) | 0 | 0 | 0 |  | 0 | 0 |  | 0 | 1 | 0 |  | 0 | 0 |
|  |  |  |  |  |  |  |  |  |  |  |  |  |  |  |
| Caecal Fat | None (0) | 43 | 95 | 90 |  | 81 | 80 |  | 19 | 86 | 81 |  | 73 | 71 |
|  | Little, moderate or excessive (1-4) | 57 | 5 | 10 |  | 19 | 20 |  | 81 | 14 | 19 |  | 27 | 29 |
|  |  |  |  |  |  |  |  |  |  |  |  |  |  |  |
| Mesenteric Fat | None (0) | 50 | 79 | 73 |  | 80 | 76 |  | 16 | 80 | 79 |  | 71 | 71 |
|  | Little, moderate or excessive (1-4) | 50 | 21 | 27 |  | 20 | 24 |  | 84 | 20 | 21 |  | 29 | 29 |
|  |  |  |  |  |  |  |  |  |  |  |  |  |  |  |
| Spleen | Red or black (0-1); Abnormal, | 100 | 90 | 91 |  | 84 | 95 |  | 66 | 83 | 89 |  | 93 | 93 |
|  | enlarged, granular or nodular (2-4) |  | 9 | 9 |  | 16 | 5 |  | 34 | 17 | 11 |  | 7 | 7 |
|  |  |  |  |  |  |  |  |  |  |  |  |  |  |  |

**Table B in S3 Appendix (continued). Summary of gross necropsy scores for yearling Chinook**.

|  |  |  | | | | | |  |  | | | | | |
| --- | --- | --- | --- | --- | --- | --- | --- | --- | --- | --- | --- | --- | --- | --- |
| Metric | Score summary | 2007 | | | | | |  | 2008 | | | | | |
|  |  | Reference | McNary Dam (225 km) | |  | Bonneville Dam (460 km) | |  | Reference | McNary Dam (225 km) | |  | Bonneville Dam (460 km) | |
|  |  |  | AT | PIT |  | AT | PIT |  |  | AT | PIT |  | AT | PIT |
| Food in Gut | Absent (0) | 87 | 91 | 87 |  | 56 | 45 |  | 86 | 91 | 91 |  | 93 | 82 |
|  | Present (1) | 13 | 9 | 13 |  | 44 | 55 |  | 14 | 9 | 9 |  | 7 | 18 |
|  |  |  |  |  |  |  |  |  |  |  |  |  |  |  |
| Hind Gut | No inflammation (0) | 100 | 97 | 100 |  | 100 | 100 |  | 100 | 96 | 93 |  | 100 | 97 |
|  | Mild or severe inflammation (1‑2) | 0 | 2 | 0 |  | 0 | 0 |  | 0 | 4 | 7 |  | 0 | 3 |
|  |  |  |  |  |  |  |  |  |  |  |  |  |  |  |
| Liver | Normal, firm, red‑brown (0) | 93 | 76 | 83 |  | 63 | 77 |  | 84 | 84 | 86 |  | 78 | 79 |
|  | Abnormal, discolored (1‑5) | 7 | 25 | 17 |  | 37 | 23 |  | 16 | 16 | 14 |  | 22 | 21 |
|  |  |  |  |  |  |  |  |  |  |  |  |  |  |  |
| Gall Bladder | Yellow, empty or partially full (0) | 25 | 8 | 20 |  | 19 | 23 |  | 0 | 22 | 14 |  | 9 | 5 |
|  | Discolored or distended (1‑3) | 75 | 93 | 80 |  | 81 | 77 |  | 100 | 78 | 86 |  | 91 | 95 |
|  |  |  |  |  |  |  |  |  |  |  |  |  |  |  |
| Kidney | Normal (0) | 100 | 97 | 97 |  | 88 | 87 |  | 100 | 100 | 100 |  | 99 | 99 |
|  | Pale or abnormalities (1‑4) | 0 | 3 | 3 |  | 13 | 13 |  | 0 | 0 | 0 |  | 1 | 1 |
|  |  |  |  |  |  |  |  |  |  |  |  |  |  |  |

^*^ Totals may exceed 100 due to rounding.

**Table C in S3 Appendix. Summary of gross necropsy results for subyearling Chinook, 2007.** Fish were recaptured at Bonneville Dam, 460 km downstream from release. Geode index scores were binned to normal vs. non-normal results.

|  |  |  |  |  |
| --- | --- | --- | --- | --- |
|  | Subyearling Chinook salmon (%) | | | |
|  |  |  |  |  |
| Metric | Geode index criterion/scale | Reference  fish  (n = 79) | Bonneville Dam recaptures | |
|  |  |  | AT | PIT |
|  |  |  | (n = 9) | (n = 71) |
| Smolt index | Fully smolted (0) | 52 | 100 | 94 |
|  | Partial, weakly or non-smolted (1‑3) | 49 | 0 | 6 |
|  |  |  |  |  |
| Eyes | Normal (0) | 100 | 100 | 100 |
|  | Abnormality or damage (1-5) | 0 | 0 | 0 |
|  |  |  |  |  |
| Fins | Normal (0) | 100 | 100 | 88 |
|  | Opaque, damaged, frayed or missing (1-3) | 0 | 0 | 12 |
|  |  |  |  |  |
| Gills | Normal (0) | 100 | 100 | 100 |
|  | Pale, marginate, or clubbed (1‑3) | 0 | 0 | 0 |
|  |  |  |  |  |
| Pseudobranchs | Normal (0) | 100 | 100 | 100 |
|  | Swollen, lithic, inflamed (1‑4) | 0 | 0 | 0 |
|  |  |  |  |  |
| Caecal fat | None (0) | 63 | 89 | 76 |
|  | Little (1) | 28 | 11 | 24 |
|  | Moderate (2) | 9 | 0 | 0 |
|  | Excessive (3) | 0 | 0 | 0 |
|  |  |  |  |  |
| Mesenteric fat | None (0) | 65 | 89 | 82 |
|  | Little (1) | 25 | 11 | 18 |
|  | Moderate (2) | 10 | 0 | 0 |
|  | Excessive (3) | 0 | 0 | 0 |
|  |  |  |  |  |
| Spleen | Red or Black (0-1) | 97 | 100 | 89 |
|  | Abnormal, enlarged, granular or nodular (2-4) | 3 | 0 | 11 |
|  |  |  |  |  |
| Food in gut | Absent (0) | 100 | 43 | 48 |
|  | Present (1) | 0 | 57 | 52 |
|  |  |  |  |  |
| Hind gut | No inflammation (0) | 100 | 100 | 99 |
|  | Mild or severe inflammation (1‑2) | 0 | 0 | 1 |
|  |  |  |  |  |
| Liver | Normal, firm, red‑brown (0) | 90 | 56 | 65 |
|  | Abnormal, discolored (1‑5) | 10 | 44 | 35 |
|  |  |  |  |  |
| Gall bladder | Yellow, empty or part full (0) | 11 | 44 | 42 |
|  | Discolored or distended (1‑4) | 89 | 55 | 58 |
|  |  |  |  |  |
| Kidney | Normal (0) | 99 | 67 | 86 |
|  | Pale or swollen (1‑4) | 1 | 33 | 14 |
|  |  |  |  |  |
