# Supplementary material for: Pushing the envelope: Micro-transmitter effects on small juvenile Chinook salmon (Oncorhynchus tshawytscha)
Source: PLoS One. 2020 Mar 25;15(3):e0230100. doi: 10.1371/journal.pone.0230100 (PMC7094837; doi:10.1371/journal.pone.0230100)
Supplement: S4 Appendix — (DOCX) [file pone.0230100.s005.docx]

**S4 Appendix: Histological evaluation**

**Methodology**

Tissues for histological analyses were taken from each fish collected for gross necropsy and placed into one of three separate cassettes labeled gill (gill), soft tissue (heart, liver, head and trunk kidney, spleen, upper and lower intestine and pyloric ceca), and incision (skin in area of incision/suture). All tissue samples were placed directly into Davidson’s solution for fixation and left undisturbed for 7‑14 d.

After fixation, tissue samples were rinsed with distilled water and transferred to 70% ethyl alcohol for continued preservation until they were processed further. Fixed tissues were dehydrated, processed using a Shandon Hypercenter XP automated tissue processor, and embedded in Polyfin (Triangle Biomedical Sciences). Tissues sections (4‑5 µm thick) were stained with haematoxylin and eosin‑phloxine (Luna 1968) and examined by light microscopy at the Ecotoxicology and Environmental Fish Health Program laboratory of the Northwest Fisheries Science Center in Seattle, WA.

**Table A in S4 Appendix. Metrics for scoring histological evaluation**. Note scales of 0‑1 indicate presence (1) or absence (0)

|  | |  |  |  |
| --- | --- | --- | --- | --- |
| Histological | |  |  |  |
| metric | |  | Definition or description | Scale |
| *Nutritional indicators* | |  |  |  |
|  | Liver vacuolation |  | Measure of the normal glycogen (energy) or lipid/fat stores in liver; primarily glycogen. | 1‑7 |
|  | Pancreatic zymogen |  | Digestive enzyme wherein low levels or absence indicates that a fish has stopped eating. | 0‑3 |
|  | Pancreatic atrophy |  | Evidence that pancreatic cells have shrunk (indicates fish has stopped eating). | 0‑1 |
|  | Mesenteric adipose |  | Fat reserves in the mesentery as a nutritional measure. | 0‑3 |
|  | pyloric caecae mucosal glycogen |  | Glycogen reserves in the pyloric caecae | 0‑1 |
|  | Small intestinal digesta presence |  | Presence of food in the small intestine. | 0‑1 |
|  | Lower intestinal mucosal glycogen |  | Glycogen stores in the lower intestine. | 0‑3 |
|  | Lower intestinal digesta presence |  | Food presence in the large intestine. | 0‑1 |
|  | Liver hydropic vacuolation |  | Water vacuoles in liver cell may indicate previous exposure to chlorinated hydrocarbons (marine fish) or change in pH. Can indicate inadequate diet in some mammals; unknown relation to diet in salmonids | 1‑7 |
|  |  |  |  |  |
| *Inflammatory indicators* | |  |  |  |
|  | Pancreatic inflammation |  | Inflammatory cell infiltrates in and around the exocrine pancreas. | 0‑1 |
|  | Small intestinal inflammation |  | Presence of intestinal inflammation. | 0‑1 |
|  | Lower intestinal inflammation |  | Inflammation in the lower intestine. | 0‑1 |
|  | Heart epi/myocarditis |  | Inflammation of either the epicardium (epicarditis) or myocardium (myocarditis) of the heart. | 0‑1 |
|  | Spleen congestion |  | Typically indicates a generalized response to stress. | 0‑1 |
|  | Spleen lymphoid depletion |  | Reduction in normal proportion of white pulp (lymphoid tissue) to red pulp (erythropoietic tissue) in the spleen. | 0‑1 |
|  | Splenic macrophage aggregates |  | Normal structures, indicating activity of reticuloendothelial system | 1‑7 |
|  | Mesenteric chronic inflammation |  | Inflammation in mesentery | 0‑1 |
|  | Mesenteric chronic inflammations |  | Severity of inflammation in mesentery | 1‑7 |
|  | Peritonitis, chronic |  | Internal adhesions at the site of the incision. When present, no obvious signs of infectious cause (e.g., large amounts of bacteria), however, could not be ruled out. | 0‑1 |
|  | |  |  |  |
| *Degenerative indicators* | |  |  |  |
|  | Liver coagulative necrosis |  | Coagulative necrosis in hepatocytes of liver | 0‑1 |
|  | Liver eosinophilic hypertrophy |  | Hepatocytes stain more eosinophilic than usual due to hypertrophy; occurrence often related to degenerative changes. | 0‑1 |
|  | Kidney tubule epithelial necrosis |  | Coagulative necrosis of the epithelium lining the tubules of the kidney nephrons. | 0‑1 |

**Table A in S4 Appendix (continued). Metrics for scoring histological evaluation**.

|  | |  |  |  |
| --- | --- | --- | --- | --- |
| Histological | |  |  |  |
| metric | |  | Definition or description | Scale |
| I*nfectious indicators/agents* | |  |  |  |
|  | Liver lymphocytic infiltrates |  | Infiltrates of mononuclear inflammatory cells and/or perivascular cuffing (aggregated around blood vessels) can indicate response to BKD/infectious agent. | 0‑1 |
|  | Liver BKD lesions |  | Lesions suggestive of bacterial kidney disease in liver. | 0‑1 |
|  | Liver Ceratomyxa lesions |  | Ceratomyxa shasta‑like myxosporeans in liver. | 0‑1 |
|  | Small intestinal digenetic trematodes |  | When present, small intestinal trematodes appeared to be at commensal levels. | 0‑1 |
|  | Small intestinal Ceratomyxa |  | Organisms resembling Ceratomyxa shasta in mucosa of small intestine. |  |
|  | Lower intestinal digenetic trematodes |  | If present, levels did not appear higher than normal. No indication that trematodes were causing problems for these fish. | 0‑1 |
|  | Kidney BKD lesions |  | Indication of a host response to BKD infection. | 0‑1 |
|  | Kidney tubule Myxosporea |  | Unidentified myxosporean infection of the epithelium lining the kidney tubules. | 0‑1 |
|  |  |  |  |  |
| *Incision/injection‑site healing* | |  |  |  |
|  | Incision closure |  | Describes whether the incision appears closed over by epidermal cells; 1 = closure, 0 = open, no closure. | 0‑1 |
|  | Skin stratum compactum reknit |  | Reknitting or reconnection of stratum compactum layer in dermis, where layer on either side of surgical incision has joined. | 0‑1 |
|  | Incision chronic inflammation |  | Chronic inflammatory infiltrates (e.g. macrophages, lymphocytes) at the incision site. | 0‑1 |
|  | Incision chronic inflammation severity |  | Degree of cellular infiltrates in region of incision | 0‑7 |
|  | Dermal muscular necrosis |  | Residual muscle necrosis at incision site. | 0‑1 |
|  | Incision, poor apposition |  | Uneven apposition between two sides of incision (i.e. overlapping closure) creates larger entry point for secondary pathogens 1 = poor 0 = good | 0‑1 |
|  | Incision, adhesions |  | Adhesions between mesenteries associated with internal organs and peritoneum at incision/suture site. Adhesions are usually associated with chronic peritonitis. | 0‑1 |
|  | Internal organ evulsion via incision; presence of Saprolegnia |  | Evaluated internally and externally | 0‑1 |
|  | Incision not visible |  |  | 0‑1 |
|  | Epidermis retracted |  |  | 0‑1 |
|  |  |  |  |  |
| *Other* | |  |  |  |
|  | Kidney tubule HYDVAC |  | Water vacuoles in the kidney tubule cells. | 0‑1 |
|  | Small intestinal mucosal glycogen |  | Glycogen reserves in the small intestine. This is generally not a good indicator of nutritional status. | 0‑3 |
|  | Spleen macrophage aggregates |  | Normal structures, indicating activity of reticuloendothelial system. | 1‑7 |
|  |  |  |  |  |

**Results: yearling Chinook salmon**

**Table B in S4 Appendix. Comparative analysis of histological exam scores for yearlings, 2007‑2008.**  Summary of significant differences by tag treatment based on histology scores for yearling Chinook recaptured at Bonneville and McNary Dam (α = 0.01). Dashes indicate no significant difference. N/O indicates metrics not observed in either group.

|  |  | | | | | | |
| --- | --- | --- | --- | --- | --- | --- | --- |
|  | Significant difference in prevalence/severity or amount (*p*‑value) | | | | | | |
|  | 2007 | | | 2008 | | | |
| Histological metric | McNary Dam | Bonneville Dam | | McNary Dam | | Bonneville/  John Day Dam | |
|  |  |  | |  | |  | |
|  | Greater in acoustic-tagged yearling Chinook | | | | | | |
| *Nutritional* |  |  | |  | |  | |
| Pancreatic zymogen | -- | ‑‑ | | ‑‑ | | 0.035 | |
| Mesenteric adipose | ‑‑ | 0.014 | | 0.003 | | ‑‑ | |
| Pyloric caecae mucosal glycogen | ‑‑ | -- | | ‑‑ | | ‑‑ | |
| Liver hydropic vacuolation | 0.006 | ‑‑ | | ‑‑ | | ‑‑ | |
| *Inflammatory* |  |  | |  | |  | |
| Spleen congestion | 0.027 | ‑‑ | | ‑‑ | | ‑‑ | |
| Mesenteric chronic inflammation | ‑‑ | ‑‑ | | 0.001 | | 0.001 | |
| Mesenteric chronic inflammation severity | ‑‑ | ‑‑ | | <0.001 | | <0.001 | |
| Peritonitis, chronic | 0.042 | 0.003 | | ‑‑ | | 0.001 | |
| Infectious indicators/agents |  |  | |  | |  | |
| Gill amoebiasis | N/O | N/O | | -- | | ‑‑ | |
| *Incision/injection site healing* |  |  | |  | |  | |
| Incision chronic inflammation severity | ‑‑ | 0.001 | | ‑‑ | | -- | |
| Incision, poor apposition | 0.000 | 0.000 | | ‑‑ | | 0.006 | |
| Incision, adhesions | 0.008 | -- | | ‑‑ | | -- | |
| Epidermis retracted | N/O | N/O | | ‑‑ | | 0.015 | |
|  |  |  | |  | |  | |
|  | Greater in PIT-tagged yearling Chinook | | | | | | |
| *Nutritional* |  |  | |  | |  | |
| Lower intestinal mucosal glycogen | ‑‑ | 0.036 | | 0.004 | | ‑‑ | |
| Lower intestinal digesta presence | 0.021 | ‑‑ | | ‑‑ | | ‑‑ | |
| *Inflammatory* |  |  | |  | |  | |
| Pancreatic inflammation | ‑‑ | 0.026 | | N/O | | N/O | |
| *Infectious indicators/agents* |  |  | |  | |  | |
| Lower intestinal digenetic trematodes | ‑‑ | ‑‑ | | ‑‑ | | 0.047 | |
| Incision/injection‑site healing |  |  | |  | |  | |
| *Incision closure* | ‑‑ | 0.011 | | ‑‑ | | ‑‑ | |
| Skin stratum compactum reknit | 0.002 | -- | | ‑‑ | | ‑‑ | |
| Incision not visible | N/O | N/O | | 0.001 | | 0.001 | |
| *Other* |  |  | |  | |  | |
| Small intestinal mucosal glycogen | ‑‑ | 0.004 | | ‑‑ | | ‑‑ | |
|  |  | |  | |  | |  |

***Nutritional indices***

In 2007, the presence of digesta in the lower intestine, a positive nutritional indicator, was higher in PIT‑tagged fish at McNary Dam, as was lower intestinal mucosal glycogen at Bonneville Dam. However, although liver hydropic vacuolization (an indicator of inadequate diet in some mammals) was higher in AT fish at McNary Dam, so was mesenteric adipose, which is a positive nutritional indicator. In 2008, lower intestinal mucosal glycogen was again rated higher in the PIT group at McNary Dam, however, mesenteric adipose was once again rated higher in the AT group. Finally, pancreatic zymogen (packets of digestive enzymes) was rated higher in the AT group at Bonneville Dam.

***Peritoneal inflammation***

Splenic congestion was rated higher in AT fish at McNary Dam in 2007. Chronic inflammation of the mesentery was rated as more prevalent and of greater severity in AT fish at both McNary and Bonneville Dams in 2008. AT fish in both 2007 recapture groups, as well as the 2008 recapture group at Bonneville Dam, exhibited a higher prevalence of chronic peritonitis than PIT fish. Peritonitis was evaluated locally, at the incision site, and was thought to have been a primary reaction to the tag. The one exception to the trend of higher inflammation in the AT group occurred at Bonneville Dam in 2007, where PIT fish were observed histologically to have a higher prevalence of pancreatic inflammation.

***Incision healing***

Microscopically, there was greater prevalence of skin stratum compactum reknitting (an indicator of tissue healing) in PIT than in AT fish at McNary Dam in 2007. Incision closure was also rated higher in the PIT group at Bonneville Dam that year. In 2008, skin stratum compactum reknitting was seen in only one fish of each tag treatment at McNary Dam, and in two PIT-tagged fish at Bonneville/John Day Dam. Poor apposition was described more often in AT than PIT fish recaptured at McNary and Bonneville Dams in 2007 as well as at Bonneville/John Day in 2008. Similarly, the epidermis was rated as retracted more often in AT than PIT fish at Bonneville in 2008. When present, the severity of chronic inflammation at the incision site was rated higher in AT fish at Bonneville Dam in 2007. AT fish were also rated as having a higher incidence of adhesions associated with the incision than PIT fish at McNary Dam in 2007. Organ evulsion was observed in one AT fish at McNary Dam in 2008.

**Results: subyearling Chinook salmon**

**Table C in S4 Appendix. Comparative analysis of scores from histological examinations of subyearlings in 2007.** Summary of significant differences (α = 0.01) of comparative analyses of histological metrics in subyearling Chinook salmon recaptured at Bonneville Dam by tag treatment, 2007.

|  |  |
| --- | --- |
| Histological metric | Significant difference in prevalence/severity or amount  (*P*‑value) |
|  |  |
|  | Greater in AT |
| Peritonitis, chronic | 0.007 |
| Mesenteric chronic inflammation severity | 0.075 |
| Incision chronic inflammation severity | 0.011 |
| Dermal hemorrhage/fibrin | 0.009 |
|  |  |
|  | Greater in PIT |
| Skin stratum compactum reknit | 0.001 |
| Small intestinal mucosal glycogen | 0.020 |
|  |  |

Subyearling AT fish had a significantly higher prevalence of chronic peritonitis than PIT fish (*P* = 0.007). Mesenteric chronic inflammation severity was also higher in AT fish (*P* = 0.075). The severity of chronic inflammation at the incision site was higher in AT fish (*P* = 0.011), as was the presence of dermal hemorrhage/fibrin (*P* = 0.009). Reknitting of the skin stratum compactum layer (i.e. healing) was higher in PIT fish (*P* = 0.001). Reserves of glycogen in the small intestine were also significantly higher in PIT fish (*P* = 0.020), though this index is not a particularly strong indicator of nutritional status.
