# Supplementary material for: Pushing the envelope: Micro-transmitter effects on small juvenile Chinook salmon (Oncorhynchus tshawytscha)
Source: PLoS One. 2020 Mar 25;15(3):e0230100. doi: 10.1371/journal.pone.0230100 (PMC7094837; doi:10.1371/journal.pone.0230100)
Supplement: S5 Appendix — (DOCX) [file pone.0230100.s006.docx]

**S5 Appendix: Laboratory holding study**

**Table A in S5 Appendix. Sample sizes for laboratory holding study, 2007‑2008.** Numbers of subsampled Chinook salmon held in the laboratory by tag treatment and life history type.

|  |  | |
| --- | --- | --- |
| Tag treatment  and life history type | Sample size (n) | |
|  | 2007 | 2008 |
|  |  |  |
| Acoustic tag |  |  |
| Yearling | 400 | 400 |
| Subyearling | 360 | 403 |
|  |  |  |
| Surgical PIT tag |  |  |
| Yearling | 0 | 400 |
| Subyearling | 0 | 400 |
|  |  |  |
| Injected PIT tag |  |  |
| Yearling | 400 | 400 |
| Subyearling | 360 | 408 |
|  |  |  |
| Reference fish (non-tagged) |  |  |
| Yearling | 400 | 400 |
| Subyearling | 360 | 405 |
|  |  |  |
| Totals | 2,640 | 3,456 |
|  |  |  |

**Table B in S5 Appendix. Ambient river temperature at tagging for laboratory replicates**.

| Replicate | Temperature  2007 (°C) | Temperature  2008 (°C) |
| --- | --- | --- |
|  | Yearling Chinook salmon | |
| 1 | 11 | 8 |
| 2 | 11 | 10 |
| 3 | 11 | 10 |
| 4 | 11 | 10 |
| 5 | 12 | 10 |
| 6 | 11 | 10 |
| 7 | 11 | 10 |
| 8 | 11 | 10 |
| 9 | 11 | 10 |
| 10 | 13 | 10 |
|  | Subyearling Chinook salmon | |
| 11 | 16 | 12 |
| 12 | 15 | 11 |
| 13 | 15 | 11 |
| 14 | 16 | 11 |
| 15 | 17 | 14 |
| 16 | 18 | 14 |
| 17 | 19 | 14 |
| 18 | 20 | 14 |
| 19 | 20 | 16 |
| 20 | 19 | 17 |

**Table C in S5 Appendix. Survival of laboratory yearlings, 2007.** Survival at 14, 28, and 90 d by tag treatment and replicate for yearling Chinook salmon held at Bonneville Dam.

|  |  | | | |
| --- | --- | --- | --- | --- |
| Treatment date | **Yearling Chinook salmon survival, 2007 (SE)** | | | |
|  | Reference | Acoustic transmitter | Passive integrated transponder |  |
|  |  |  |  |  |
|  | **14 d holding** | | | |
| 24 Apr | 0.95 (0.03) | 0.95 (0.03) | 0.95 (0.03) |  |
| 25 Apr | 0.90 (0.05) | 0.88 (0.05) | 0.97 (0.03) |  |
| 27 Apr | 0.95 (0.03) | 0.88 (0.05) | 0.98 (0.02) |  |
| 30 Apr | 0.68 (0.08) | 0.79 (0.07) | 0.84 (0.05) |  |
| 2 May | 0.95 (0.05) | 0.63 (0.011) | 0.74 (0.07) |  |
| 4 May | 0.98 (0.02) | 0.95 (0.03) | 1.00 (0.00) |  |
| 7 May | 0.88 (0.05) | 0.73 (0.07) | 0.83 (0.06) |  |
| 9 May | 0.98 (0.02) | 0.93 (0.04) | 0.93 (0.04) |  |
| 11 May | 1.00 (0.00) | 0.85 (0.06) | 0.95 (0.03) |  |
| 14 May | 1.00 (0.00) | 0.92 (0.04) | 0.98 (0.02) |  |
| Total | 0.92 (0.02) | 0.86 (0.02) | 0.92 (0.02) |  |
| Mean | 0.93 (0.02) | 0.85 (0.02) | 0.92 (0.02) |  |
|  |  |  |  |  |
|  | **28 d holding** | | | |
| 24 Apr | 0.95 (0.03) | 0.93 (0.04) | 0.95 (0.03) |  |
| 25 Apr | 0.85 (0.06) | 0.88 (0.05) | 0.92 (0.04) |  |
| 27 Apr | 0.95 (0.03) | 0.85 (0.06) | 0.98 (0.02) |  |
| 30 Apr | 0.66 (0.08) | 0.74 (0.07) | 0.82 (0.06) |  |
| 2 May | 0.84 (0.08) | 0.53 (0.011) | 0.74 (0.07) |  |
| 4 May | 0.98 (0.02) | 0.95 (0.03) | 1.00 (0.00) |  |
| 7 May | 0.79 (0.06) | 0.70 (0.07) | 0.75 (0.07) |  |
| 9 May | 0.95 (0.03) | 0.88 (0.05) | 0.88 (0.05) |  |
| 11 May | 0.97 (0.03) | 0.83 (0.06) | 0.93 (0.04) |  |
| 14 May | 1.00 (0.00) | 0.87 (0.05) | 0.95 (0.03) |  |
| Total | 0.89 (0.02) | 0.83 (0.02) | 0.89 (0.02) |  |
| Mean | 0.89 (0.02) | 0.81 (0.02) | 0.89 (0.02) |  |
|  |  |  |  |  |
|  | **90 d holding** | | | |
| 24 Apr | 0.88 (0.05) | 0.75 (0.07) | 0.88 (0.05) |  |
| 25 Apr | 0.75 (0.07) | 0.70 (0.07) | 0.38 (0.08) |  |
| 27 Apr | 0.77 (0.07) | 0.60 (0.08) | 0.88 (0.05) |  |
| 30 Apr | 0.45 (0.08) | 0.63 (0.08) | 0.49 (0.07) |  |
| 2 May | 0.68 (0.011) | 0.32 (0.11) | 0.44 (0.08) |  |
| 4 May | 0.78 (0.07) | 0.70 (0.07) | 0.95 (0.03) |  |
| 7 May | 0.57 (0.08) | 0.45 (0.08) | 0.63 (0.08) |  |
| 9 May | 0.90 (0.05) | 0.73 (0.07) | 0.85 (0.06) |  |
| 11 May | 0.79 (0.07) | 0.70 (0.07) | 0.85 (0.06) |  |
| 14 May | 0.82 (0.07) | 0.85 (0.06) | 0.93 (0.04) |  |
| Total | 0.74 (0.02) | 0.66 (0.02) | 0.72 (0.02) |  |
| Mean | 0.74 (0.04) | 0.64 (0.04) | 0.73 (0.04) |  |
|  |  |  |  |  |

**Table D in S5 Appendix. Survival of laboratory yearlings, 2008.** Survival at 14, 28 and 120 d by tag treatment and replicate group of yearlings held at Bonneville Dam.

|  |  |  |  |  |  |
| --- | --- | --- | --- | --- | --- |
| Treatment date | **Yearling Chinook salmon survival, 2008 (SE)** | | | |  |
|  |  |  |  |  |  |
|  | Reference | Injectable passive integrated transponder | Surgical passive integrated transponder | Surgical acoustic transmitter |  |
|  |  |  |  |  |  |
|  | **14 d holding** | | | |  |
| 23 Apr | 1.00 (0.00) | 1.00 (0.00) | 0.88 (0.05) | 0.85 (0.06) |  |
| 28 Apr | 0.93 (0.04) | 0.98 (0.02) | 1.00 (0.00) | 0.93 (0.04) |  |
| 30 Apr | 0.90 (0.05) | 1.00 (0.00) | 0.88 (0.05) | 0.83 (0.06) |  |
| 2 May | 0.87 (0.05) | 0.85 (0.06) | 0.77 (0.07) | 0.82 (0.06) |  |
| 5 May | 0.83 (0.06) | 0.78 (0.07) | 0.43 (0.09) | 0.68 (0.08) |  |
| 7 May | 0.87 (0.05) | 0.80 (0.06) | 0.72 (0.07) | 0.87 (0.05) |  |
| 9 May | 0.67 (0.08) | 0.72 (0.07) | 0.76 (0.07) | 0.84 (0.06) |  |
| 12 May | 0.85 (0.06) | 0.86 (0.05) | 0.95 (0.04) | 0.83 (0.06) |  |
| 14 May | 0.78 (0.07) | 0.84 (0.06) | 0.82 (0.06) | 0.95 (0.04) |  |
| 16 May | 0.83 (0.06) | 0.90 (0.05) | 0.87 (0.05) | 0.93 (0.04) |  |
| Mean | 0.85 (0.03) | 0.87 (0.03) | 0.81 (0.05) | 0.85 (0.02) |  |
|  |  |  |  |  |  |
|  |  |  |  |  |  |
|  | **28 d holding** | | | | |
| 23 Apr | 0.95 (0.04) | 1.00 (0.00) | 0.88 (0.05) | 0.73 (0.07) |  |
| 28 Apr | 0.90 (0.05) | 0.98 (0.02) | 1.00 (0.00) | 0.90 (0.05) |  |
| 30 Apr | 0.90 (0.05) | 1.00 (0.00) | 0.83 (0.06) | 0.80 (0.06) |  |
| 2 May | 0.84 (0.06) | 0.83 (0.06) | 0.77 (0.07) | 0.71 (0.07) |  |
| 5 May | 0.73 (0.07) | 0.72 (0.07) | 0.37 (0.09) | 0.63 (0.08) |  |
| 7 May | 0.79 (0.06) | 0.76 (0.07) | 0.59 (0.08) | 0.82 (0.06) |  |
| 9 May | 0.56 (0.08) | 0.62 (0.08) | 0.65 (0.08) | 0.76 (0.07) |  |
| 12 May | 0.79 (0.06) | 0.81 (0.06) | 0.85 (0.06) | 0.78 (0.07) |  |
| 14 May | 0.75 (0.07) | 0.82 (0.06) | 0.79 (0.06) | 0.95 (0.04) |  |
| 16 May | 0.73 (0.07) | 0.86 (0.05) | 0.77 (0.07) | 0.88 (0.05) |  |
| Mean | 0.80 (0.04) | 0.84 (0.04) | 0.75 (0.06) | 0.80 (0.03) |  |
|  |  |  |  |  |  |
|  |  | | | | |
|  | **120 d holding** | | | | |
| 23 Apr | 0.87 (0.05) | 0.97 (0.03) | 0.83 (0.06) | 0.61 (0.08) |  |
| 28 Apr | 0.80 (0.06) | 0.83 (0.06) | 0.85 (0.06) | 0.76 (0.07) |  |
| 30 Apr | 0.75 (0.07) | 0.95 (0.03) | 0.70 (0.07) | 0.68 (0.07) |  |
| 2 May | 0.76 (0.07) | 0.60 (0.08) | 0.74 (0.07) | 0.63 (0.08) |  |
| 5 May | 0.61 (0.08) | 0.72 (0.07) | 0.37 (0.09) | 0.55 (0.08) |  |
| 7 May | 0.74 (0.07) | 0.71 (0.07) | 0.51 (0.08) | 0.77 (0.07) |  |
| 9 May | 0.49 (0.08) | 0.59 (0.08) | 0.46 (0.08) | 0.71 (0.07) |  |
| 12 May | 0.77 (0.07) | 0.63 (0.07) | 0.74 (0.07) | 0.68 (0.07) |  |
| 14 May | 0.58 (0.08) | 0.63 (0.08) | 0.67 (0.08) | 0.82 (0.06) |  |
| 16 May | 0.63 (0.08) | 0.76 (0.07) | 0.62 (0.08) | 0.66 (0.07) |  |
| Mean | 0.70 (0.04) | 0.74 (0.04) | 0.65 (0.05) | 0.69 (0.03) |  |
|  |  |  |  |  |  |

**Table E in S5 Appendix. Overall growth of laboratory yearlings, 2007.** Mean increases in length (mm) at 90 d by tag treatment for replicates groups of tagged yearling Chinook held in the laboratory, Bonneville Dam, 2007. Standard errors in parentheses.

|  |  | |
| --- | --- | --- |
| Treatment  date | **Yearling Chinook salmon growth over 90 days , 2007 (mm)** | |
|  | Acoustic transmitter | Passive integrated transponder |
| 24 Apr | 36.5 (2.5) | 37.9 (2.3) |
| 25 Apr | 36.1 (2.1) | 40.2 (2.7) |
| 27 Apr | 30.5 (2.9) | 41.9 (1.5) |
| 30 Apr | 31.0 (2.9) | 41.2 (2.6) |
| 2 May | 40.0 (5.7) | 33.8 (2.5) |
| 4 May | 27.5 (2.1) | 35.4 (1.8) |
| 7 May | 30.2 (3.7) | 35.7 (1.9) |
| 9 May | 32.8 (2.3) | 34.2 (2.2) |
| 11 May | 35.6 (2.3) | 33.2 (1.7) |
| 14 May | 34.3 (2.5) | 36.9 (2.1) |
| Mean | 33.4 (1.2) | 37.1 (1.2) |
|  |  |  |

**Table F in S5 Appendix. Overall growth of laboratory yearlings 2008.** Mean increases in length (mm) and weight (g) at 120 d by tag treatment and replicate for yearling Chinook salmon held in the laboratory, Bonneville Dam, 2008. Standard errors in parentheses.

|  |  | | |
| --- | --- | --- | --- |
| Treatment  date | **Yearling Chinook salmon growth over 120 days, 2008 (mm)** | | |
|  | Injectable passive integrated transponder | Surgical passive integrated transponder | Surgical acoustic transmitter |
|  |  | | |
|  |  |  |  |
|  | Mean increase in length (mm) | | |
| 23 Apr | 73.79 (2.51) | 72.03 (2.77) | 69.96 (4.13) |
| 28 Apr | 67.76 (3.54) | 70.66 (2.84) | 63.16 (2.95) |
| 30 Apr | 63.08 (2.33) | 53.63 (2.72) | 51.54 (3.51) |
| 2 May | 59.13 (3.64) | 65.29 (4.9)**^a^** | 63.04 (4.65) |
| 5 May | 56.73 (3.16) | 69.64 (5.12) | 66.24 (3.71) |
| 7 May | 57.31 (3.22) | 62.85 (4.28) | 65.93 (1.88) |
| 9 May | 67.52 (2.37) | 69.76 (3.9) | 59.11 (3.49) |
| 12 May | 55.37 (3.33) | 54.28 (2.71) | 54.89 (3.84) |
| 14 May | 59.67 (2.77) | 49.15 (3.11) | 57.00 (2.96) |
| 16 May | 60.81 (3.01) | 58.25 (3.47) | 55.70 (3.48) |
| Mean | 62.67 (1.01) | 62.34 (1.22) | 60.51 (1.16) |
|  |  |  |  |
|  |  |  |  |
|  | Mean increase in weight (g) | | |
| 23 Apr | NA (0) | 89.60 (3.66) | 83.14 (5.3) |
| 28 Apr | NA (0) | 87.73 (4.04) | 81.04 (3.55) |
| 30 Apr | 81.49 (3.65) | 76.90 (5.11) | 77.77 (5.52) |
| 2 May | 73.26 (5.45) | 75.69 (6.76)**^c^** | 73.93 (5.94) |
| 5 May | 81.66 (5.03)**^b^** | 85.96 (8.13) | 84.82 (6.56) |
| 7 May | 76.76 (5.34) | 91.82 (5.82)**^d^** | 84.75 (2.56) |
| 9 May | 85.60 (3.29) | 102.98 (5.8) | 77.70 (6.36)**^e^** |
| 12 May | 73.84 (4.93) | 74.68 (4.38) | 75.04 (7.46) |
| 14 May | 76.22 (3.64) | 65.76 (5.43) | 82.91 (5.56) |
| 16 May | 80.39 (5.87) | 77.10 (6.65)**^f^** | 73.51 (5.62)**^g^** |
| Mean | 78.77 (1.72) | 82.99 (1.79) | 79.19 (1.81) |
|  |  |  |  |
|  |  |  |  |

^a^ N = 28, ^b^ N = 23, ^c^ N = 28, ^d^ N = 19, ^e^ N = 26, ^f^ N = 17, ^g^ N = 26

**Table G in S5 Appendix. Survival of laboratory subyearlings, 2007**. Estimated survival by tag treatment and replicate at 14, 28, and 90 d holding for subyearling Chinook.

|  | | | |
| --- | --- | --- | --- |
| **Subyearling Chinook salmon survival, 2007 (SE)** | | | |
| Treatment Date | Reference | Acoustic transmitter | Passive integrated transponder |
|  | **14 d holding** | | |
| 6 Jun | 0.98 (0.02) | 0.92 (0.04) | 1.00 (0.00) |
| 12 Jun | 0.98 (0.02) | 0.68 (0.07) | 1.00 (0.00) |
| 15 Jun | 0.97 (0.03) | 0.38 (0.08) | 0.95 (0.03) |
| 19 Jun | 0.95 (0.03) | 0.43 (0.08) | 0.97 (0.03) |
| 21 Jun | 0.80 (0.06) | 0.45 (0.08) | 0.98 (0.02) |
| 26 Jun | 0.95 (0.03) | 0.68 (0.07) | 0.93 (0.04) |
| 28 Jun | 0.87 (0.05) | 0.73 (0.07) | 0.98 (0.02) |
| 5 Jul | 0.78 (0.07) | 0.39 (0.08) | 0.87 (0.05) |
| 11 Jul | 0.73 (0.07) | 0.13 (0.05) | 0.83 (0.06) |
| Total | 0.89 (0.02) | 0.53 (0.03) | 0.94 (0.01) |
| Mean | 0.88 (0.04) | 0.53 (0.04) | 0.94 (0.04) |
|  | **28 d holding** | | |
| 6 Jun | 0.95 (0.03) | 0.69 (0.07) | 0.95 (0.04) |
| 12 Jun | 0.98 (0.02) | 0.51 (0.08) | 0.95 (0.04) |
| 15 Jun | 0.82 (0.06) | 0.28 (0.07) | 0.90 (0.05) |
| 19 Jun | 0.93 (0.04) | 0.35 (0.08) | 0.95 (0.04) |
| 21 Jun | 0.73 (0.07) | 0.28 (0.07) | 0.95 (0.03) |
| 26 Jun | 0.95 (0.03) | 0.61 (0.08) | 0.86 (0.05) |
| 28 Jun | 0.79 (0.06) | 0.58 (0.08) | 0.98 (0.02) |
| 5 Jul | 0.68 (0.07) | 0.32 (0.08) | 0.85 (0.06) |
| 11 Jul | 0.51 (0.08) | 0.10 (0.05) | 0.60 (0.08) |
| Total | 0.81 (0.02) | 0.41 (0.03) | 0.89 (0.02) |
| Mean | 0.82 (0.03) | 0.41 (0.03) | 0.89 (0.03) |
|  | **90 d holding** | | |
| 6 Jun | 0.88 (0.05) | 0.62 (0.08) | 0.92 (0.04) |
| 12 Jun | 0.93 (0.04) | 0.46 (0.08) | 0.85 (0.06) |
| 15 Jun | 0.59 (0.08) | 0.15 (0.06) | 0.80 (0.06) |
| 19 Jun | 0.83 (0.06) | 0.35 (0.08) | 0.87 (0.05) |
| 21 Jun | 0.68 (0.07) | 0.18 (0.06) | 0.83 (0.06) |
| 26 Jun | 0.85 (0.06) | 0.46 (0.08) | 0.74 (0.07) |
| 28 Jun | 0.72 (0.07) | 0.45 (0.08) | 0.98 (0.02) |
| 5 Jul | 0.58 (0.08) | 0.21 (0.07) | 0.67 (0.08) |
| 11 Jul | 0.37 (0.08) | 0.08 (0.04) | 0.33 (0.07) |
| Total | 0.71 (0.02) | 0.33 (0.02) | 0.77 (0.02) |
| Mean | 0.71 (0.04) | 0.33 (0.04) | 0.77 (0.04) |
|  |  |  |  |

**Table H in S5 Appendix. Survival of laboratory subyearlings, 2008**. Estimated survival by tag treatment and replicate group for subyearling Chinook held at 14, 28, and 120 d.

| Treatment date | |  | | | |
| --- | --- | --- | --- | --- | --- |
|  |  | **Subyearling Chinook salmon survival, 2008 (SE)** | | | |
|  |  | Reference | Injectable passive integrated transponder | Surgical passive integrated transponder | Surgical acoustic transmitter |
|  | | **14 d holding** | | | |
| 3 Jun | | 1.00 (0.00) | 1.00 (0.00) | 1.00 (0.00) | 1.00 (0.00) |
| 5 Jun | | 0.93 (0.04) | 1.00 (0.00) | 1.00 (0.00) | 1.00 (0.00) |
| 10 Jun | | 0.97 (0.03) | 1.00 (0.00) | 0.98 (0.02) | 0.95 (0.03) |
| 12 Jun | | 0.98 (0.02) | 1.00 (0.00) | 1.00 (0.00) | 0.90 (0.05) |
| 18 Jun | | 0.98 (0.02) | 1.00 (0.00) | 0.97 (0.03) | 0.90 (0.05) |
| 20 Jun | | 1.00 (0.00) | 1.00 (0.00) | 0.95 (0.04) | 0.93 (0.04) |
| 25 Jun | | 0.98 (0.02) | 0.97 (0.03) | 0.90 (0.05) | 0.84 (0.06) |
| 27 Jun | | 0.95 (0.04) | 0.98 (0.02) | 0.93 (0.04) | 0.83 (0.06) |
| 1 Jul | | 0.86 (0.05) | 0.95 (0.03) | 0.82 (0.06) | 0.88 (0.05) |
| 10 Jul | | 0.79 (0.07) | 0.78 (0.07) | 0.22 (0.07) | 0.26 (0.07) |
| Mean | | **0.94 (0.02)** | **0.97 (0.02)** | **0.88 (0.08)** | **0.85 (0.07)** |
|  | | **28 d holding** | | | |
| 3 Jun | | 1.00 (0.00) | 1.00 (0.00) | 1.00 (0.00) | 1.00 (0.00) |
| 5 Jun | | 0.90 (0.05) | 1.00 (0.00) | 0.97 (0.03) | 1.00 (0.00) |
| 10 Jun | | 0.95 (0.04) | 0.95 (0.04) | 0.98 (0.02) | 0.90 (0.05) |
| 12 Jun | | 0.98 (0.02) | 0.98 (0.02) | 1.00 (0.00) | 0.88 (0.05) |
| 18 Jun | | 0.93 (0.04) | 0.97 (0.03) | 0.97 (0.03) | 0.90 (0.05) |
| 20 Jun | | 1.00 (0.00) | 1.00 (0.00) | 0.92 (0.04) | 0.85 (0.06) |
| 25 Jun | | 0.95 (0.03) | 0.97 (0.03) | 0.87 (0.05) | 0.79 (0.07) |
| 27 Jun | | 0.90 (0.05) | 0.89 (0.05) | 0.83 (0.06) | 0.75 (0.07) |
| 1 Jul | | 0.62 (0.07) | 0.83 (0.06) | 0.72 (0.07) | 0.80 (0.06) |
| 10 Jul | | 0.71 (0.07) | 0.70 (0.08) | 0.19 (0.06) | 0.23 (0.07) |
| Mean | | **0.89 (0.04)** | **0.93 (0.03)** | **0.85 (0.08)** | **0.81 (0.07)** |
|  | **120 d holding** | | | | |
| 3 Jun | 0.76 (0.07) | | 0.88 (0.06) | 0.87 (0.06) | 0.83 (0.06) |
| 5 Jun | 0.25 (0.07) | | 0.45 (0.08) | 0.32 (0.08) | 0.15 (0.06) |
| 10 Jun | 0.79 (0.06) | | 0.56 (0.08) | 0.53 (0.08) | 0.73 (0.07) |
| 12 Jun | 0.90 (0.05) | | 0.85 (0.06) | 0.90 (0.05) | 0.83 (0.06) |
| 18 Jun | 0.63 (0.07) | | 0.79 (0.06) | 0.79 (0.06) | 0.62 (0.08) |
| 20 Jun | 0.90 (0.05) | | 0.85 (0.06) | 0.82 (0.06) | 0.78 (0.07) |
| 25 Jun | 0.74 (0.07) | | 0.77 (0.07) | 0.62 (0.08) | 0.63 (0.08) |
| 27 Jun | 0.67 (0.08) | | 0.66 (0.07) | 0.75 (0.07) | 0.65 (0.08) |
| 1 Jul | 0.31 (0.07) | | 0.69 (0.07) | 0.36 (0.08) | 0.45 (0.08) |
| 10 Jul | 0.68 (0.08) | | 0.70 (0.08) | 0.14 (0.06) | 0.21 (0.06) |
| Mean | **0.66 (0.07)** | | **0.72 (0.04)** | **0.61 (0.08)** | **0.59 (0.08)** |
|  |  | |  |  |  |

**Table I in S5 Appendix. Growth of laboratory subyearlings, 2007.** Mean increases in length (mm) and weight (g) at 90 d by tag treatment and replicate for subyearling Chinook salmon held in the laboratory. The difference in overall mean length of 4.5 mm between treatment groups was nearly significant (*P* = 0.061), while the difference in average weight gain of 3.4 g was not.

|  |  | |
| --- | --- | --- |
| Treatment  date | **Subyearling Chinook salmon growth over 90 days, 2007** | |
|  | AT (SE) | PIT (SE) |
|  |  |  |
|  | Mean increase in length (mm) | |
| 6 Jun | 28.2 (11.2) | 33.3 (9.2) |
| 12 Jun | 31.3 (8.0) | 27.6 (9.7) |
| 15 Jun | 40.0 (11.4) | 34.6 (15.0) |
| 19 Jun | 36.6 (11.1) | 40.0 (8.0) |
| 21 Jun | 25.3 (13.3) | 35.5 (5.3) |
| 26 Jun | 32.4 (13.2) | 36.1 (10.7) |
| 28 Jun | 27.8 (14.1) | 33.8 (9.5) |
| 5 Jul | 26.4 (11.7) | 32.2 (9.4) |
| 11 Jul | 17.7 (17.7) | 32.9 (17.8) |
| Mean | 29.5 (1.5) | 34.0 (1.5) |
|  |  |  |
|  | Mean increase in weight (g) | |
| 6 Jun | 19.2 (9.6) | 20.3 (6.8) |
| 12 Jun | 22.3 (8.2) | 20.6 (8.4) |
| 15 Jun | 33.0 (16.6) | 20.6 (7.3) |
| 19 Jun | 23.4 (5.7) | 28.5 (8.6) |
| 21 Jun | 17.0 (9.6) | 27.5 (6.9) |
| 26 Jun | 23.3 (11.2) | 25.4 (9.6) |
| 28 Jun | 20.9 (11.3) | 26.0 (8.9) |
| 5 Jul | 21.0 (11.7) | 24.5 (9.9) |
| 11 Jul | 10.3 (10.1) | 27.8 (21.8) |
| Mean | 21.2 (1.9) | 24.6 (1.9) |
|  |  |  |

**Table J in S5 Appendix. Growth of laboratory subyearlings, 2008.** Overall mean growth in length (mm) and weight (g) at 120 d by tag treatment and replicate for subyearling Chinook salmon.

|  |  |  | |  | |
| --- | --- | --- | --- | --- | --- |
| Treatment date | **Subyearling Chinook salmon growth over 120 days, 2008 (SE)** | | | | |
|  |  |  | |  | |
|  | Passive integrated transponder | | | Acoustic transmitter | |
|  | Injectable | Surgical | |  |  |
|  |  |  | |  | |
|  | **Mean increase in length (mm)** | | | | |
|  |  |  | |  | |
| 3 Jun | 72.30 (2.01) | 70.35 (2.59)^a^ | | 69.64 (2.02)^b^ | |
| 5 Jun | 73.29 (2.89) | 80.00 (2.23) | | 81.50 (4.41) | |
| 10 Jun | 64.40 (2.22) | 68.35 (2.59)^c^ | | 70.40 (1.61) | |
| 12 Jun | 72.43 (1.75) | 66.19 (1.8) | | 63.76 (1.42) | |
| 18 Jun | 65.00 (1.68) | 58.13 (1.35) | | 55.79 (1.79) | |
| 20 Jun | 65.49 (1.78) | 63.88 (1.82) | | 61.81 (1.95) | |
| 25 Jun | 65.17 (2.7) | 66.54 (1.84) | | 61.33 (2.21) | |
| 27 Jun | 67.89 (2.06) | 70.2 (2.27) | | 70.58 (1.82) | |
| 1 Jul | 62.79 (3.31) | 66.64 (4.43) | | 64.22 (3.33) | |
| 10 Jul | 64.73 (3.2) | 71.2 (4.28) | | 64.38 (5.39) | |
| Mean | 67.25 (0.77) | 66.86 (0.8) | | 65.28 (0.78) | |
|  |  |  | |  | |
|  | **Mean increase in weight (g)** | | | | |
|  |  |  | |  | |
| 3 Jun | 68.00 (3.11) | 72.13 (3.61) | | 65.41 (3.14) | |
| 5 Jun | 70.91 (4.29) | 86.78 (6.02) | | 93.95 (5.41) | |
| 10 Jun | 60.1 (3.35) | 64.6 (3.85) | | 70.52 (2.83)^d^ | |
| 12 Jun | 72.39 (3.00) | 70.5 (2.37) | | 67.42 (2.37) | |
| 18 Jun | 66.13 (2.97) | 63.5 (1.93) | | 61.28 (2.67) | |
| 20 Jun | 59.62 (2.96) | 59.32 (2.72) | | 56.77 (2.95) | |
| 25 Jun | 69.4 (3.92) | 64.93 (2.37) | | 54.67 (2.92) | |
| 27 Jun | 63.53 (2.68) | 62.55 (2.91) | | 64.27 (2.35) | |
| 1 Jul | 57.44 (4.11) | 62.09 (4.91) | | 63.09 (4.71) | |
| 10 Jul | 61.91 (4.00) | 63.82 (7.94) | | 60.66 (7.82) | |
|  |  |  | |  | |
| Mean | 64.83 (1.12) | 66.2 (1.11) | | 63.85 (1.17) | |
|  |  | |  | |  |

^a^ N = 26, ^b^ N = 28, ^c^ N = 20, ^d^ N = 28
