# Supplementary material for: Pushing the envelope: Micro-transmitter effects on small juvenile Chinook salmon (Oncorhynchus tshawytscha)
Source: PLoS One. 2020 Mar 25;15(3):e0230100. doi: 10.1371/journal.pone.0230100 (PMC7094837; doi:10.1371/journal.pone.0230100)
Supplement: S6 Appendix — (DOCX) [file pone.0230100.s007.docx]

**S6 Appendix: Evaluation of disease prevalence**

Fresh kidney samples were excised and placed into individually labeled sample bags (Nasco Whirlpak, 2 oz, #B01064). Samples were frozen and transported on ice to the Northwest Fisheries Science Center. In the laboratory, kidney samples were thawed, diluted in 0.01‑M phosphate‑buffered saline with 0.05% Tween 20 at 1:4 (w/v), homogenized using a print roller, and then frozen in screw cap tubes.

For each treatment and release/laboratory group combination, the Rs antigen was determined based on enzyme‑linked immunosorbent assay (ELISA) as described by Pascho and Mulcahy (1987) and modified by Pascho et al. (1991). Coating and conjugate antibodies (Kirkegaard and Perry Laboratories, Gaithersburg MD) were used at dilutions of 1:1500 and 1:4000 respectively. Optical densities were read at 405 nm using an automated 96‑well absorbance microplate reader (Model ELx808 IU; BioTek Instruments, Inc., Winooski, VT). Negative controls and blanks, as well as substrate and conjugate controls, were run for each assay. ELISA values were reported as absolute readings, without subtracting values for blanks or negative controls.

Values obtained from ELISA testing represented an index of the magnitude of *Rs* bacteria present, and absolute values were not functionally related (e.g. the difference between 0.08 and 0.09 did not correspond to the difference between 2.5 and 2.7 via a mathematical function). Therefore, to construct metrics for measuring levels of BKD, it was prudent to map values with an indexing system to more robustly represent “distance” between ELISA values. We mapped values following the method of Pascho et al. (1991), who categorized infection based on the detection of *Rs* antigen using values of <0.199 as reflecting a low level of infection, 0.2 to 0.999 as a medium level, and values equal to or greater than 1.0 as indicating a high level of infection.
